# Global Prevalence of Fuchs Endothelial Corneal Dystrophy (FECD) in Adult Population: A Systematic Review and Meta-Analysis

**DOI:** 10.1155/2022/3091695

**Published:** 2022-04-14

**Authors:** Francesco Aiello, Gabriele Gallo Afflitto, Francesca Ceccarelli, Massimo Cesareo, Carlo Nucci

**Affiliations:** ^1^Ophthalmology Unit, Department of Experimental Medicine, University of Rome Tor Vergata, Via Montpellier 1, Rome 00133, Italy; ^2^McKnght Vision Research Centre, Bascom Palmer Eye Institute, University of Miami, Miami, FL, USA

## Abstract

**Purpose:**

To evaluate the global prevalence of Fuchs endothelial corneal dystrophy (FECD).

**Design:**

Systematic review and meta-analysis.

**Methods:**

A systematic electronic literature search was conducted on PubMed/MedLine, Cochrane Library, and Google Scholar, in order to select papers analysing the prevalence rate of FECD. Two authors independently conducted the electronic search. After removal of duplicates, title and abstract screening, and full-text analysis, data from selected articles were archived in a customized Excel spreadsheet. Risk of bias assessment was performed using the Joanna Briggs Institute Prevalence Critical Appraisal Tool. Meta-analysis was conducted using R (version 1.4.1106, package “meta”).

**Results:**

A total of 6660 eligible articles were retrieved from the initial electronic search. Only 4 original works were included in the qualitative and quantitative analysis. Of the 4746 patients included in this meta-analysis (i.e., 2232 male (M) and 2322 female (F)), we retrieved 269 FECD cases (81 M; 188 F), with a pooled prevalence estimates of 7.33% (95% CI: 4.08–12.8%). Statistically significant gender-related differences in the prevalence of FECD emerged by the analysis (OR: 2.22; 95% CI: 1.66–2.96, *p*=0.0016). While the total number of people aged >30 years with FECD is nowadays estimated at nearly 300 million, an increase of 41.7% in the number of FECD-affected patients is expected by 2050, when the overall figure is supposed to rise up to 415 million.

**Conclusion:**

This study provides a reliable figure of the present and future epidemiological burden of FECD globally, which might be useful for the design of FECD screening, treatment, rehabilitation, and related public health strategies.

## 1. Introduction

Fuchs endothelial corneal dystrophy (FECD) is a bilateral disease of the corneal endothelium. It is characterized by a progressive and accelerated loss of corneal endothelial cells accompanied by a number of degenerative processes of the Descemet membrane (DM) [1]. This primarily includes the accumulation of an aberrant extracellular matrix (ECM) and the formation of posterior focal excrescences called guttae [1, 2]. Changes in quantity and quality of vision can eventually result due to the aforementioned DM changes as well as to the disruption of the corneal endothelial pump function, leading to corneal oedema, bullae formation, and late subepithelial fibrosis [2, 3].

While two different types of FECD exist, the late-onset form represents the most common, which is usually inherited in an autosomal dominant fashion with variable penetrance and expressivity [1, 2]. In addition, numerous ophthalmic and systemic conditions have been described to variably correlate with the presence of FECD (i e., hearing loss, cardiovascular disease, keratoconus, ocular hypertension, and macular drusen), whose expression has been eventually reported to be more common in the female gender [1, 2]. However, the evaluation of the global epidemiologic features of the disease has been rendered overtly tough, due to the vast heterogeneity in the available prevalence estimates of the disease [4–7].

Thus, the aim of this meta-analysis is to estimate the cumulative global prevalence rate of FECD in the adult population. Our analysis will use the latest publicly available data to even predict the number of actual and future FECD-affected patients worldwide.

## 2. Materials and Methods

This study was conducted in strict compliance with the Preferred Reporting Items for Systematic Reviews and Meta-Analysis (PRISMA) guidelines supplementary (S0) [8]. Neither institutional review board approval nor informed consent were required for this study, since all the reported data were obtained from the available published literature. The review protocol was submitted, revised, accepted, and published by the International Prospective Register of Systematic Reviews (PROSPERO) (ID: CRD42021284423).

### 2.1. Inclusion and Exclusion Criteria

The PICOS framework was used in developing the literature search strategy [9]. Specifically, the PICOS scheme was structured as follows: patients (P), male and female adults worldwide (>30 years); investigated condition (I), FECD defined as the presence of corneal guttae by slit lamp and/or by specular microscopy examination; comparator (C), healthy control; outcome (O), prevalence rate; study type (S), randomized controlled trials and large observational studies (i e., both prospective and retrospective).

Studies were excluded if they (a) were not in English, (b) were not available in full-text form, (c) < 70% of included patient assessments were directly performed by the investigators, (d) represented a subgroup analysis of patients from a larger study, (e) the article type was either a conference abstract, a review, a case report, a book chapter, or a letter to the editor. No publication date was imposed, but articles had to be published in a peer-reviewed journal.

### 2.2. Data Source and Study Searching

An electronic search was performed on PubMed/MEDLINE, Cochrane Library, and Google Scholar using relevant keywords, phrases, and medical subject headings (MeSH) terms. The search strategy applied for both databases was: “Fuchs Dystrophy” AND “prevalence.” The “cited by” tool on Google Scholar was used to minimize the risk of missing relevant papers. The reference list of each selected article was checked to screen for additional potentially relevant studies (i e., snowballing method). The last search was carried out on December 1, 2021.

### 2.3. Data Extraction

Two reviewers independently conducted the electronic literature search (F.C. and F.A.). The reference lists from the 3 databases (i e., PubMed/MEDLINE, Cochrane Library, and Google Scholar) were merged and the duplicates removed using the reference management software EndNote X9 (version X9.3.3). Titles and abstracts of the remaining papers were screened. Whenever appropriate, the full texts of relevant articles underwent subsequent evaluation for eligibility. In the presence of eventual discrepancies, a third reviewer (C.N.) was consulted to solve the conundrum.

Per each study, the following outcome measures were retrieved: author and year of publication; country of origin; total number of screened subjects; number of FECD patients; and corresponding demographic features including age and sex. Data extracted from selected papers were archived by two independent reviewers (F.C. and F.A.) in a customized Excel (Microsoft Corp, Seattle, Washington, USA) spreadsheet with forced choice entry criteria. Corresponding authors of related articles were contacted in an attempt to obtain missing data. Whenever any outcome measure was not available, the relative study was excluded from the pooled analysis for that endpoint.

### 2.4. Risk of Bias and Study Quality Assessment

The Joanna Briggs Institute Prevalence Critical Appraisal Tool (JBI-PCAT) was used to evaluate the quality of the included studies by 3 reviewers (F.C., F.A., and G.G.A.) [10]. As recently proposed by the Prevalence Estimates Review–Systematic Review Methodology Group (PERSyst), the JBI-PCAT represents the most appropriate tool in assessing the methodological quality of prevalence studies [11].

### 2.5. Data Synthesis and Statistical Analysis

The analysis was performed using the R software for statistical computing (R 1.4.1106; “meta” package). Cochran's-Q was calculated as a measure of heterogeneity and checked by *p* value. We also reported I^2^ statistic results, which quantify heterogeneity regardless of the number of included studies. Due to the high level of expected heterogeneity, the random-effects model was used, whose results are presented on forest plot graphs.

The maximum-likelihood estimator was used to estimate the between-study variance (*τ*^2^).

Influence analysis was performed using the “InfluenceAnalysis” function in R, and a Baujat plot was consequently created.

Logit transformation (PLOGIT) of the data and a random intercept logistic regression model (GLMM) were carried out for the analysis of overall proportions, which were expressed in association with a 95% Clopper–Pearson confidence interval. Statistical significance was defined as *p* < 0.01.

According to the continent in which the study had been conducted, the included studies were classified into 5 groups: America, Asia, Europe, Africa, and Antarctica.

The population projection figures were retrieved from the United Nation World Population Prospects (UNWPP) [12], which consist of the latest results of national population surveys from countries worldwide and consider mortality and fertility rates in its projection of world population numbers. The estimated numbers of FECD patients were calculated by multiplying the age- and region-specific prevalence from our random-effects model and the corresponding population number.

## 3. Results

### 3.1. Electronic Database Search Results

**Figure 1:**
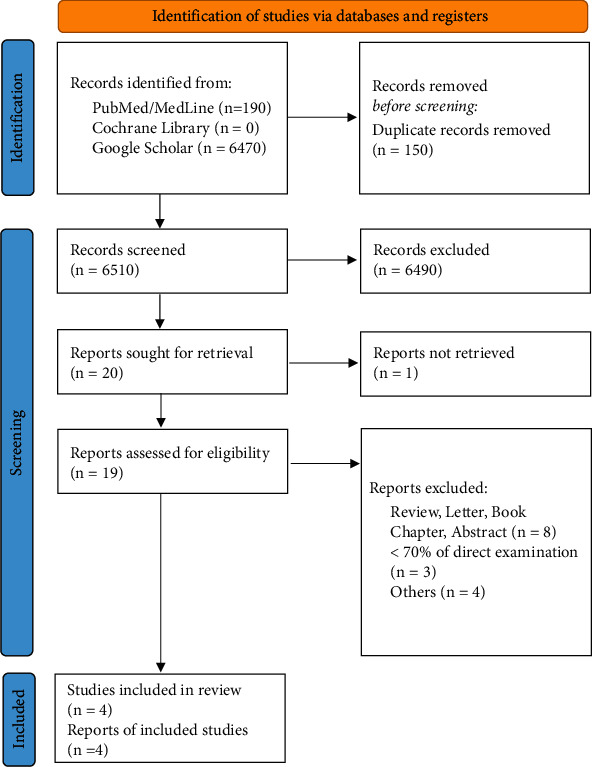
Preferred Reporting Items for Systematic Review and Meta-Analysis flowchart. Reasons for exclusion are step-by-step reported on the right.

Overall, 6660 eligible papers (i e., 190 from PubMed/MedLine, 0 from Cochrane Library, and 6470 from Google Scholar) were retrieved from the preliminary search on electronic databases. Once the duplicates had been automatically removed and both titles and abstracts were screened, 20 full-text manuscripts were assessed for eligibility being considered appropriate for the present meta-analysis. Four articles were finally included in the qualitative and in the quantitative analysis, being conformed to the aforementioned eligibility criteria (Figure 1) [4–7]. The reasons justifying the exclusion of 16 studies are reported in Figure 1. The full list of the included studies and their general features are reported in Table 1.

### 3.2. Methodological Quality and Risk of Bias of Included Studies

A moderate-to-high quality of the included studies was generally evaluated by using the JBI-PCAT tool, as shown in supplementary S1. Globally, the studies showed an unclear description of the randomization protocol and an imprecise description of the recruited sample.

### 3.3. General Features of the Analysed Population

Globally, 4746 patients were included in this meta-analysis, of whom 2232 males (M) and 2322 female (F) (M/F = 1/1) (Table 1). All included studies (100%) provided data regarding mean age (standard deviation), which was globally assessed to be as high as 61.9 years old (95% CI: 58.8–65.2). Similarly, the gender-specific FECD prevalence figures were reported by the analysed papers, while the age-specific prevalence data could have been extracted by 2 studies only [4, 5]. In addition, one of the included studies [7] was found to evaluate FECD prevalence rates in 2 geographically distinct regions. Hence, we decided to split the results according to the different populations analysed, to provide a more accurate analysis and to simplify the presentation of the results.

### 3.4. FECD Prevalence Rate

**Figure 2:**
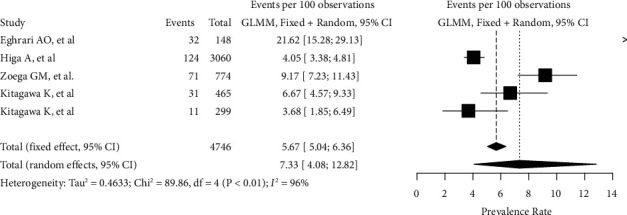
Forest plot reassuming the pooled estimate of Fuchs Endothelial Corneal Dystrophy prevalence rate. Both fixed and random-effects models are represented. GLMM, generalized linear mixed model.

Overall, 269 patients in our sample were found to be affected by FECD (81 males; 188 females). Globally, FECD prevalence rate, as assessed by this analysis, peaked up to 7.33% (95% CI: 4.08–12.8%). The heterogeneity variance among different studies was estimated at *τ*^2^ = 0.463, with an I^2^ value of 95.5% (95% CI 92.2%–97.5%). Pooled results are reported in the forest plot presented in Figure 2. Age-weighted prevalence rates are summarized in Table 2. The funnel plot generated, which shows a high asymmetry, is shown in supplementary S2. The Peter's test was not run because of the low number of studies included [13]. Results deriving from the Baujat plot and the sensitivity analysis are reported in supplementary S3 and in supplementary S4.

### 3.5. Gender and Geographical Variation

**Figure 3:**
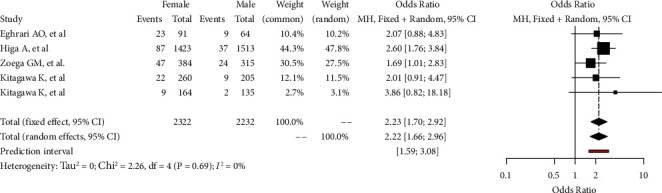
Gender-related prevalence rate of Fuchs endothelial corneal dystrophy in the adult population (>30 years old). Odds ratio is calculated and both fixed and random-effects models are represented. GLMM, generalized linear mixed model.

Among the included articles, 4/4 (100%) reported FECD prevalence data by gender. Prevalence rates of FECD in male and female were registered to be as high as 4.58 (95% CI: 2.37–8.66) (supplementary S5) and 9.84 (95% CI: 5.95–15.83) (supplementary S6), respectively. In the included studies, female gender appears to be more commonly affected by FECD than male. Statistically significant gender-related differences in the prevalence of FECD emerged from the analysis (OR: 2.22; 95% CI: 1.66–2.96, *p*=0.0016) (Figure 3).

Meaningful differences emerged from the analysis of prevalence data differences according to geographical localization, with the American continent featuring the highest prevalence rate of FECD (supplementary S7).

### 3.6. Number of People with FECD Worldwide from 2020 to 2050

As per the prevalence rate of FECD obtained by our random-effects model (7.33% (95% CI: 4.08–12.8%)) and the corresponding population number reported by the UNWPP data in 2020 (i e., more than 4 billion), the total number of people aged >30 years with FECD is estimated at nearly 300 million. However, an increase of 41.7% in the number of people (aged >30 years) with FECD is expected by 2050, when the overall figure is supposed to rise up to 415 million.

## 4. Discussion

To the best of our knowledge, this work represents the first meta-analysis trying to ascertain the global prevalence of FECD in the adult population. Specifically, it is intended to provide comprehensive, up-to-date estimations on the current global FECD prevalence as well as to forecast projection figures of the number of FECD-affected patients in 2050.

Overall, we unfortunately found a modest number of epidemiological studies on the subject, the majority of whom were conducted in Asia. A full and representative coverage of all countries was not achieved. While this evidence substantially affects the reliability of our results, it must be also considered that our work, representing the first meta-analysis on the topic, is the only available one trying to define the effective worldwide prevalence and epidemiological burden of the disease.

Overall, we estimated the global prevalence of FECD to be as high as 7.33%, with the highest figures reported in North America, where the prevalence rate of the disease is reported to peak up to 21.62% (supplementary S7). However, this result is eventually biased by the specific setting chosen by Eghrari et al. in their study [4]. In fact, they conducted their analysis in Tangier, an island in Virginia, with a population of over 500 related individuals [4]. Hence, the overtly major prevalence of the disease in such a context might be easily explained by the highly conserved pedigree of the selected population as well as considering the genetic inheritance pattern of FECD [1, 2]. Coherently, the influence analysis shows Eghrari et al.'s study to substantially contribute to the overall heterogeneity of the proposed results, which appears to not reside when the same report is removed from the pooled analysis (supplementary S4). These data strongly suggest that external modifiable and unmodifiable external factors are mainly responsible for the vast evidenced heterogeneity. As an example, the diverse genetic background specific for different population might eventually explain the possibility of regional variation in the number of FECD-affected patients, of whom few is known due to the paucity of available large epidemiologic studies on the topic.

With a total of 4 studies and more than four thousand pooled patients, our model was sufficiently powered to detect a difference between gender- and age-groups. In fact, our finding provides substantial evidence that females have a double the risk to develop FECD than the counterpart. Furthermore, we demonstrated that the odds for FECD tended to increase by a 1.2 factor when moving from the 30–50 to the 50–70 age group. Both these data are in absolute accordance with both the available genetic and pathophysiological mechanisms responsible for FECD genesis and development [1, 2]. In fact, as reported by Liu et al. in a murine in vivo model of FECD, the greater susceptibility of females than males to the development of the disease might be at least explained by the higher levels of oestrogen DNA adducts in the former, responsible for the blockage of mitochondrial both metabolic and replicative processes [14, 15].

The number of people with FECD worldwide (>30 years) will increase from 300 million in 2020 to 415 million in 2050. This mainly results from the expected growth in the number of aged people, which is anticipated to variably affect all continents. In fact, while the United Nations probabilistic projections report only minor variations in the global amount of elderly people in Europe and in North America, the same population group is expected to increase more dramatically in Asia and in Africa because of the increased life expectancy in these regions [12]. Unfortunately, due to the modest number of studies included in this meta-analysis, we believed it was not useful to try to ascertain regional variation in the expected figures of FECD.

The strengths of our meta-analysis include a critical appraisal of study quality by the rigorously validated JBI-PCAT, strict application of inclusion and exclusion criteria and the application of a statistical significance criterium of 0.01 for a more conservative approach to the proposed results. Of note, only studies with a direct examination operated by the investigators >70% were included. Unfortunately, a reasonable coverage of all world regions was not possible, due to the spurious number of large epidemiological studies on the topic. A vast intercase heterogeneity eventually derived, which is in line with different other systematic reviews and meta-analysis of prevalence [15, 16]. Second, we excluded not-in-English publications in this review. Nevertheless, all not-in-English publications did not meet our inclusion criteria. Thus, exclusion of such publications is unlikely to result in a significant publication bias in our analysis.

Finally, in our projection of FECD numbers, the overall prevalence of the disease was assumed to remain constant over time. Nevertheless, the change of prevalence over time is difficult to quantify as it depends on changes of risk exposure and other external factors, such as public awareness of the condition, screening campaign, and diagnostic technological improvements which might in turn modify the clinical approach to the condition. As a fact, the recent implementation of deep learning algorithms has highlighted the potential of these tools in identifying early FECD cases, based on the analysis of one anterior segment-optical coherence scan without additional imaging modalities (e g., pachymetry, specular microscopy, and confocal microscopy) or other information [16]. The future adoption of such software in clinical practice might in turn determine an increase in the number of people with a diagnosis of FECD, due to the higher sensitivity of our diagnostic toolkit.

In conclusion, our study provides estimates that reflect the present and future burden of FECD globally. The findings of our analysis might be useful for the design of FECD screening, treatment, rehabilitation, and related public health strategies worldwide.

## Figures and Tables

**Table 1:** General features of the articles included in the qualitative and quantitative analysis.SD, standard deviation; FECD, Fuchs Endothelial Corneal Dystrophy; USA United States of America.

Author	Age (mean ± SD)	FECD patients	Patients (total)	FECD (male)	Male (total)	FECD (female)	Female (total)	Country
Eghrari et al. [4]	57	32	148	9	64	23	91	USA (Tangier island)
Higa et al. [5]	59.1 ± 14.9	124	3060	37	1513	87	1423	Japan
Zoega et al. [6]	70	71	774	24	315	47	384	Iceland
Kitagawa et al. [7]	62.1 ± 7.6	31	465	9	205	22	260	Singapore
Kitagawa et al. [7]	64.4 ± 8.1	11	299	2	135	9	164	Japan

**Table 2:** Age-weighted prevalence rates of Fuchs endothelial corneal dystrophy.

Group	Age (years)	No. of studies	FECD prevalence (%)	95% CI (%)
1	<50	2	7.17	1.79–24.70
2	50 to 69	2	9.20	2.40–29.47
3	>70	2	10.92	4.64–23.63

No., number; FECD, Fuchs endothelial corneal dystrophy; CI, confidence interval.

## Data Availability

Previously reported data were used to support this study and are cited at relevant places within the text.

## Supplementary Materials

Supplementary Materials Supplementary S0: PRISMA guidelines checklist. Specific page and lines are outlined per each specific item. Supplementary S1: risk of bias assessment according to the Joanna Briggs Institute Prevalence Critical Appraisal tool. (a) Was the sample frame appropriate to address the target population? (b) Were study participants recruited in an appropriate way? (c) Was the sample size adequate? (d) Were the study subjects and setting described in detail? (e) Was data analysis conducted with sufficient coverage of the identified sample? (f) Were valid methods used for the identification of the condition? (g) Was the condition measured in a standard, reliable way for all participants? (h) Was there appropriate statistical analysis? (i) Was the response rate adequate? and if not, was the low response rate managed appropriately? Supplementary S2: color-enhanced funnel plot demonstrating a marked asymmetry. Supplementary S3: Baujat plot representing single-study influence analysis on pooled results. Supplementary S4: results obtained by the influence analysis. Only a slight reduction in heterogeneity derives from removal of highly influent studies. FECD, Fuchs endothelial corneal dystrophy; CI, confidence interval. Supplementary S5: prevalence rate of Fuchs endothelial corneal dystrophy in the adult male population (>30 years old). Both fixed and random-effects models are represented. GLMM, generalized linear mixed model. Supplementary S6: prevalence rate of Fuchs endothelial corneal dystrophy in the adult female population (>30 years old). Both fixed and random effect models are represented. GLMM, generalized linear mixed model. Supplementary S7: geographic variation of Fuchs endothelial corneal dystrophy prevalence rate. As evident, an unequal distribution of studies across the 5 continents exists. Both fixed and random-effects models are represented. GLMM, generalized linear mixed model.
