# Supplementary material for: Global Prevalence of Fuchs Endothelial Corneal Dystrophy (FECD) in Adult Population: A Systematic Review and Meta-Analysis
Source: J Ophthalmol. 2022 Apr 14;2022:3091695. doi: 10.1155/2022/3091695 (PMC9023201; doi:10.1155/2022/3091695)
Supplement: Supplementary Materials — Supplementary S0: PRISMA guidelines checklist. Specific page and lines are outlined per each specific item. Supplementary S1: risk of bias assessment according to the Joanna Briggs Institute Prevalence Critical Appraisal tool. (a) Was the sample frame appropriate to address the target population? (b) Were study participants recruited in an appropriate way? (c) Was the sample size adequate? (d) Were the study subjects and setting described in detail? (e) Was data analysis conducted with sufficient coverage of the identified sample? (f) Were valid methods used for the identification of the condition? (g) Was the condition measured in a standard, reliable way for all participants? (h) Was there appropriate statistical analysis? (i) Was the response rate adequate? and if not, was the low response rate managed appropriately? Supplementary S2: color-enhanced funnel plot demonstrating a marked asymmetry. Supplementary S3: Baujat plot representing single-study influence analysis on pooled results. Supplementary S4: results obtained by the influence analysis. Only a slight reduction in heterogeneity derives from removal of highly influent studies. FECD, Fuchs endothelial corneal dystrophy; CI, confidence interval. Supplementary S5: prevalence rate of Fuchs endothelial corneal dystrophy in the adult male population (>30 years old). Both fixed and random-effects models are represented. GLMM, generalized linear mixed model. Supplementary S6: prevalence rate of Fuchs endothelial corneal dystrophy in the adult female population (>30 years old). Both fixed and random effect models are represented. GLMM, generalized linear mixed model. Supplementary S7: geographic variation of Fuchs endothelial corneal dystrophy prevalence rate. As evident, an unequal distribution of studies across the 5 continents exists. Both fixed and random-effects models are represented. GLMM, generalized linear mixed model. [file 3091695.f1.zip › 3091695.f1/S0.docx]

| **Section and Topic** | **Item #** | **Checklist item** | **Location where item is reported** |
| --- | --- | --- | --- |
| **TITLE** | | |  |
| Title | 1 | Identify the report as a systematic review. | Pag1. Line2 |
| **ABSTRACT** | | |  |
| Abstract | 2 | See the PRISMA 2020 for Abstracts checklist. | Pag2. Line24-46 |
| **INTRODUCTION** | | |  |
| Rationale | 3 | Describe the rationale for the review in the context of existing knowledge. | Pag3. Line52-68 |
| Objectives | 4 | Provide an explicit statement of the objective(s) or question(s) the review addresses. | Pag3. Line69-70 |
| **METHODS** | | |  |
| Eligibility criteria | 5 | Specify the inclusion and exclusion criteria for the review and how studies were grouped for the syntheses. | Pag4. Line81-91 |
| Information sources | 6 | Specify all databases, registers, websites, organisations, reference lists and other sources searched or consulted to identify studies. Specify the date when each source was last searched or consulted. | Pag4. Line94-100 |
| Search strategy | 7 | Present the full search strategies for all databases, registers and websites, including any filters and limits used. | Pag4. Line94-100 |
| Selection process | 8 | Specify the methods used to decide whether a study met the inclusion criteria of the review, including how many reviewers screened each record and each report retrieved, whether they worked independently, and if applicable, details of automation tools used in the process. | Pag4. Line103-109 |
| Data collection process | 9 | Specify the methods used to collect data from reports, including how many reviewers collected data from each report, whether they worked independently, any processes for obtaining or confirming data from study investigators, and if applicable, details of automation tools used in the process. | Pag4. Line110-118 |
| Data items | 10a | List and define all outcomes for which data were sought. Specify whether all results that were compatible with each outcome domain in each study were sought (e.g. for all measures, time points, analyses), and if not, the methods used to decide which results to collect. | Pag5. Line110-118 |
|  | 10b | List and define all other variables for which data were sought (e.g. participant and intervention characteristics, funding sources). Describe any assumptions made about any missing or unclear information. | Pag5. Line110-118 |
| Study risk of bias assessment | 11 | Specify the methods used to assess risk of bias in the included studies, including details of the tool(s) used, how many reviewers assessed each study and whether they worked independently, and if applicable, details of automation tools used in the process. | Pag5. Line121-125 |
| Effect measures | 12 | Specify for each outcome the effect measure(s) (e.g. risk ratio, mean difference) used in the synthesis or presentation of results. | Pag6. Line138-141 |
| Synthesis methods | 13a | Describe the processes used to decide which studies were eligible for each synthesis (e.g. tabulating the study intervention characteristics and comparing against the planned groups for each synthesis (item #5)). |  |
|  | 13b | Describe any methods required to prepare the data for presentation or synthesis, such as handling of missing summary statistics, or data conversions. | Pag5. Line110-118 |
|  | 13c | Describe any methods used to tabulate or visually display results of individual studies and syntheses. | Pag6. Line 129-141 |
|  | 13d | Describe any methods used to synthesize results and provide a rationale for the choice(s). If meta-analysis was performed, describe the model(s), method(s) to identify the presence and extent of statistical heterogeneity, and software package(s) used. | Pag6. Line 129-141 |
|  | 13e | Describe any methods used to explore possible causes of heterogeneity among study results (e.g. subgroup analysis, meta-regression). | Pag6. Line 129-141 |
|  | 13f | Describe any sensitivity analyses conducted to assess robustness of the synthesized results. | Pag6. Line 129-141 |
| Reporting bias assessment | 14 | Describe any methods used to assess risk of bias due to missing results in a synthesis (arising from reporting biases). | Pag6. Line 129-141 |
| Certainty assessment | 15 | Describe any methods used to assess certainty (or confidence) in the body of evidence for an outcome. | Pag6. Line 129-141 |
| **RESULTS** | | |  |
| Study selection | 16a | Describe the results of the search and selection process, from the number of records identified in the search to the number of studies included in the review, ideally using a flow diagram. | Pag7. Line 153-160 |
|  | 16b | Cite studies that might appear to meet the inclusion criteria, but which were excluded, and explain why they were excluded. | Pag7. Line 153-160. Figure 1 |
| Study characteristics | 17 | Cite each included study and present its characteristics. | Pag7. Line 153-160. Table 1 |
| Risk of bias in studies | 18 | Present assessments of risk of bias for each included study. | S1 |
| Results of individual studies | 19 | For all outcomes, present, for each study: (a) summary statistics for each group (where appropriate) and (b) an effect estimate and its precision (e.g. confidence/credible interval), ideally using structured tables or plots. | Figure 2-3; S6-7 |
| Results of syntheses | 20a | For each synthesis, briefly summarise the characteristics and risk of bias among contributing studies. | Page 8. Line 180-198 Figure 2-3; S6-7 |
|  | 20b | Present results of all statistical syntheses conducted. If meta-analysis was done, present for each the summary estimate and its precision (e.g. confidence/credible interval) and measures of statistical heterogeneity. If comparing groups, describe the direction of the effect. | Page 8. Line 180-198 Figure 2-3; S6-7 |
|  | 20c | Present results of all investigations of possible causes of heterogeneity among study results. | S2-4 |
|  | 20d | Present results of all sensitivity analyses conducted to assess the robustness of the synthesized results. | S2-4 |
| Reporting biases | 21 | Present assessments of risk of bias due to missing results (arising from reporting biases) for each synthesis assessed. |  |
| Certainty of evidence | 22 | Present assessments of certainty (or confidence) in the body of evidence for each outcome assessed. | Page 8. Line 180-198 Figure 2-3; S6-7 |
| **DISCUSSION** | | |  |
| Discussion | 23a | Provide a general interpretation of the results in the context of other evidence. | Page 9-11. Line 209-251 |
|  | 23b | Discuss any limitations of the evidence included in the review. | Page 9-11. Line 251-268 |
|  | 23c | Discuss any limitations of the review processes used. | Page 9-11. Line 251-268 |
|  | 23d | Discuss implications of the results for practice, policy, and future research. | Page11. Line 269-271 |
| **OTHER INFORMATION** | | |  |
| Registration and protocol | 24a | Provide registration information for the review, including register name and registration number, or state that the review was not registered. | Pag4. Line 76-78 |
|  | 24b | Indicate where the review protocol can be accessed, or state that a protocol was not prepared. | Pag4. Line 76-78 |
|  | 24c | Describe and explain any amendments to information provided at registration or in the protocol. | NA |
| Support | 25 | Describe sources of financial or non-financial support for the review, and the role of the funders or sponsors in the review. | Pag12. Line273-276 |
| Competing interests | 26 | Declare any competing interests of review authors. | Pag12. Line273-276 |
| Availability of data, code and other materials | 27 | Report which of the following are publicly available and where they can be found: template data collection forms; data extracted from included studies; data used for all analyses; analytic code; any other materials used in the review. |  |

*From:*  Page MJ, McKenzie JE, Bossuyt PM, Boutron I, Hoffmann TC, Mulrow CD, et al. The PRISMA 2020 statement: an updated guideline for reporting systematic reviews. BMJ 2021;372:n71. doi: 10.1136/bmj.n71

For more information, visit: <http://www.prisma-statement.org/>
