# Supplementary material for: Global Prevalence of Fuchs Endothelial Corneal Dystrophy (FECD) in Adult Population: A Systematic Review and Meta-Analysis
Source: J Ophthalmol. 2022 Apr 14;2022:3091695. doi: 10.1155/2022/3091695 (PMC9023201; doi:10.1155/2022/3091695)
Supplement: Supplementary Materials — Supplementary S0: PRISMA guidelines checklist. Specific page and lines are outlined per each specific item. Supplementary S1: risk of bias assessment according to the Joanna Briggs Institute Prevalence Critical Appraisal tool. (a) Was the sample frame appropriate to address the target population? (b) Were study participants recruited in an appropriate way? (c) Was the sample size adequate? (d) Were the study subjects and setting described in detail? (e) Was data analysis conducted with sufficient coverage of the identified sample? (f) Were valid methods used for the identification of the condition? (g) Was the condition measured in a standard, reliable way for all participants? (h) Was there appropriate statistical analysis? (i) Was the response rate adequate? and if not, was the low response rate managed appropriately? Supplementary S2: color-enhanced funnel plot demonstrating a marked asymmetry. Supplementary S3: Baujat plot representing single-study influence analysis on pooled results. Supplementary S4: results obtained by the influence analysis. Only a slight reduction in heterogeneity derives from removal of highly influent studies. FECD, Fuchs endothelial corneal dystrophy; CI, confidence interval. Supplementary S5: prevalence rate of Fuchs endothelial corneal dystrophy in the adult male population (>30 years old). Both fixed and random-effects models are represented. GLMM, generalized linear mixed model. Supplementary S6: prevalence rate of Fuchs endothelial corneal dystrophy in the adult female population (>30 years old). Both fixed and random effect models are represented. GLMM, generalized linear mixed model. Supplementary S7: geographic variation of Fuchs endothelial corneal dystrophy prevalence rate. As evident, an unequal distribution of studies across the 5 continents exists. Both fixed and random-effects models are represented. GLMM, generalized linear mixed model. [file 3091695.f1.zip › 3091695.f1/S1 (1).docx]

| **Author** | **Sample Frame^a^** | **Randomization^b^** | **Sample size^c^** | **Description^d^** | **Coverage bias^e^** | **Outcome Measures^f^** | **Data Collection^g^** | **Statistical Analysis^h^** | **Response Rate^i^** | **RoB** |
| --- | --- | --- | --- | --- | --- | --- | --- | --- | --- | --- |
| Eghrari AO, et al ^4^ | Yes | Unclear | Yes | Yes | Yes | No | Yes | Yes | Yes | **Moderate** |
| Higa A, et al ^5^ | Yes | NA | Yes | Yes | Yes | Yes | Yes | Yes | Yes | **Low** |
| Zoega GM, et al. ^6^ | Yes | Yes | Yes | Unclear | Yes | Yes | Yes | Yes | Yes | **Low** |
| Kitagawa K, et al ^7^ | Yes | Unclear | Yes | Unclear | Yes | Unclear | Yes | Yes | Yes | **Moderate** |
| Kitagawa K, et al ^7^ | Yes | Unclear | Yes | Unclear | Yes | Unclear | Yes | Yes | Yes | **Moderate** |
